# Understanding how young people transitioning from out-of-home care acquire and develop independent living skills and knowledge: A systematic review of longitudinal studies

**DOI:** 10.1371/journal.pone.0304965

**Published:** 2024-06-11

**Authors:** Michael Starr, Reinie Cordier, Eduwin Pakpahan, Matthew Robinson, Renée Speyer, Donna Chung

**Affiliations:** 1 Department of Social Work, Education and Community Wellbeing, Northumbria University, Newcastle, United Kingdom; 2 School of Allied Health, Curtin University, Perth, Western Australia, Australia; 3 Department of Health and Rehabilitation Sciences, Faculty of Health Sciences, University of Cape Town, Cape Town, South Africa; 4 Applied Statistics Research Group, Department of Mathematics, Physics and Electrical Engineering, Northumbria University, Newcastle, United Kingdom; 5 Psychological Therapies and Mental Health, Leeds Beckett University, Leeds, United Kingdom; 6 Department of Special Needs Education, University of Oslo, Oslo, Norway; 7 Department of Otorhinolaryngology and Head and Neck Surgery, Leiden University Medical Centre, Leiden, The Netherlands; Bar-Ilan University, ISRAEL

## Abstract

Young people leaving state care often experience hardship in many areas of their life. At a population level, their outcomes in early adulthood are poorer compared to general populations. Effective preparation for leaving care and post-care support systems is vital to improving outcomes. Individual and systemic support for young people to acquire Independent Living Skills (ILS) in the following eight ILS domains have been identified: Financial Management, Knowledge of Accessing Available Supports, Managing Housing, Education Planning, Job Seeking, Health Risk Management, Domestic and Self-help Task, and Managing Relationships. This systematic review aims to identify, summarise, and appraise longitudinal studies that address ILS across these ILS domains to understand better how outcomes could be improved. Seven databases (CINAHL, Embase, ProQuest, PsychINFO, PubMed, Scopus, and Web of Science) were searched on 20^th^ July 2023. In total, twenty-seven studies published between 1994 and 2022 from various countries met the eligibility criteria. The included studies reported on 2–4 waves and adopted different methodological approaches. Study quality was scored using Qualsyst. Study characteristics and details of the interventions are presented in tables. Studies cover overlapping ILS domains, which are mapped in a matrix. Results revealed that nearly three-quarters (74% or 20 out of 27) of studies explored four or fewer of the eight ILS domains. The most frequent ILS domain covered was ‘Knowledge of Accessing Available Supports’ (19/27 studies). The main conclusion considers the concept of independence as a misnomer, with ILS covering multiple, intersecting, and interdependent domains, which ultimately help and hinder one another. Further research is required to adopt a more comprehensive approach encompassing all the domains to better inform policy, programs, and practice. A limitation is that a meta-analysis was not conducted for this review. This study registered a ‘Protocol’ with OSF Registries (DOI: 10.17605/OSF.IO/MJ3ZX) on June 5^th^, 2022.

## Introduction

### The transition from out-of-home care

There is consistent international evidence that indicates that as a population and across the life course, young people who have been in the care of the state before entering adulthood have poorer outcomes compared with peers in the general population [1, 2]. A better understanding of how to improve experiences and outcomes is crucial because the number of children and young people entering out-of-home care (OOHC) is increasing across jurisdictions. For example, there were 43,100 children in care [3] in Australia between 2018–2019, and the following year the number of children in care was reported to be approximately 45,000 (a nearly 4% rise) [4]. England saw a comparable 7% increase in the children in care population over a three-year timeframe (75,420 in 2018 and 80,850 in 2021) [5, 6].

The terms used to describe children and young people in this population have varied over time and across jurisdictions. Children in the care of the state are sometimes referred to as ‘looked-after-children’ [6], ‘children-in-care’ [7] or children in ‘out-of-home care’ [8]. Children preparing to leave care, or who are transitioning from care, generally from age 15 and older, are sometimes referred to as young people exiting care or as ‘care leavers’ [9]. Similarly, those who have formally left care are often referred to as care leavers. More recently, the term ‘care-experienced’ has been used to refer to adults who were in state care at some point in their childhood [10]. Generally, ‘care-experienced’ refers to a child looked after away from their parent(s), such as in foster care, residential care, secure care, semi-independent care, kinship care, and children who have been adopted. Whilst outcomes are more likely to be poorer, the entire care-experienced population is not a homogenous group, given that experiences pre-, during, and post-care vary substantially.

Therefore, transition from care policies, systems of response, and programs are of great importance because they can potentially influence outcomes across the entire life course for care-experienced people. Transitions at multiple points across the life course are universal [11], however, for children and young people in, and preparing to leave, OOHC the transition experience is more complex as taken-for-granted supports may not be in place. Young people leaving care may have to contend with rapid or ‘instant adulthood’ [12]. Therefore, transitions from care may feel rushed and be ‘accelerated’ [9]. Many care leavers are forced to comprehend and handle more significant life changes and challenges in a shorter timeframe, with potentially fewer quality relationships to support them, which can contribute to poorer outcomes and expectations. Interdependence as a concept can offer an approach that embraces a more fluid transition to adulthood, holding in mind the value in relationships as ‘safety-nets’, and that young people are dependent and independent at the same time [13].

Care-experienced people have faced significant and impactful changes from childhood onwards, resulting in transition times that may trigger survival strategies [9, 12]. Exiting care is an ‘important transition point’ [14], given the pressures of coping with the combined environmental and emotional responsibilities of practising to live independently [15]. Parsons, Chung [16] describe the leaving care experience as ‘a major life transformation’, highlighting the process of shifting from dependency on state care to so-called ‘independence and self-reliance’, which may be interpreted as no longer requiring support.

### The recurring international theme of poor outcomes

Transition from care research consistently highlights how care leavers experience poorer outcomes across countries internationally, such as research from Australia [17–20], Belgium [21], China [22], Ethiopia [23], Ghana [24], India [25], Israel [26], Scandinavia [27], South Africa [28, 29], the UK [9, 12, 30], and the USA [31–34]. The complex interplay of factors contributing to adverse outcomes means that positive outcomes can only be achieved by supporting young people across multiple Independent Living Skills (ILS) domains. For example, limited education is likely to result in poorly paid work or unemployment which, in turn, places people at greater risk of being unable to maintain appropriate housing. This highlights the interconnectedness between economic and social structures and the complexity of constructing appropriate types of supports care-experienced young people need.

The challenges experienced by young people during the transition from care have been noted across what Powers, Geenen [35] describe as a ‘substantial body of research’. Additionally, the ‘contextual and interpersonal factors’ [9] faced during this time are even more striking when considering how these young people ‘fare systematically worse’ [27] comparatively to general populations. Previous reviews that explored transitioning from care, independent living programmes and ILS have highlighted young people’s levels of preparedness, both psychologically and practically, for developing skills that support positive independent living in early adulthood post-care. For young people during the transition from care stage, having ILS enables better outcomes across different domains. ILS acquisition generally requires support from people, systems, and ILS programmes (i.e., interdependency) [13].

Some international reviews have explored the effectiveness of ILS programmes and presented practice guidance for developing such programmes [36]. Cassarino-Perez, Córdova [37] and O’Donnell, Hatzikiriakidis [4] conducted reviews that found interventions largely facilitated improvements in care leavers’ ILS outcomes by having various supports in place, as well as young people having the capacity to make decisions about their lives. A review by Everson-Hock, Jones [14] raised questions about the level of reliable evidence relating to the success and effectiveness of specific ILS programmes, highlighting variability across their findings, which is likely to reflect that the programmes differ in scope and reach, making them difficult to compare.

‘Relationships and resilience’ is a conspicuous theme in other reviews. For example, Häggman-Laitila, Salokekkilä [38] highlight how young people leaving care struggle with independence if they have nobody to rely on, which, for example, could include mentors [21]. Reliable relationships can create a sense of (and actual) stability, which can, in turn, support independence preparation and the acquisition of new knowledge in developing ILS [39]. Another theme is ‘preparedness’, for example, Häggman-Laitila, Salokekkilä [40] and Doucet, Greeson [41] noted the lack of focus on ILS for young people while they are still in care, which led to young people feeling unprepared once they left care. Some other reviews broadly explored how ILS support different life domain outcomes, for example, Kääriälä and Hiilamo [27] identified nine different life outcomes. Whereas Woodgate, Morakinyo [42] noted the positive impact ILS has on housing, education, and employment as a result of young people’s participation in special ILS programmes, such as transitional housing programs where there is a focus on independent living across the whole life, or where interventions focused on mentorship and onsite education or, employment specific support services.

### Importance of ILS and knowledge for ‘moving on’

Young people entering the transition from care period often have reported they were ‘ill-equipped for independent living’ [43]. From the age of 15 to 25 years, when young people enter ‘emerging adulthood’ [44], they require dependable relationships that are personally relevant to them [38, 45, 46]. Moreover, young people need to develop a strong sense of self [35, 37] to support their individual ability to acquire the ILS they need to move forward in life. Care-experienced young people may have to navigate added challenges such as managing health conditions, exiting juvenile justice systems, finding affordable, stable housing, and a multitude of associated hardships.

All children, whether they have been in care or not, need to develop skills as they mature that can help them learn responsibility and become confident and prepared to manage tasks in life. The care system attempts to establish reliable relationships and attachments, which can create developmentally appropriate dependencies, which is especially important at transition points where greater exposure to experiences may build skills. When young people age out of the care system, the focus shifts to ‘independence’ and self-sufficiency [47]. The idea of independence in child protection stems from legislation, whereby the state is responsible until the person reaches the legal age of adulthood (generally at age eighteen), when rights and responsibilities change. The concept of independence is attached to this responsibility. However, such responsibilities are not demanded of those who are not within the child protection system. Lived experiences can impact individuals’ skills and knowledge acquisition in different ways and at different times, which are often not considered systemically for those leaving care. In reality, skills are developed at points when needed, as well as through trial and error, and can be understood as an ability to ‘know’ or put knowledge into practice. The concept of independence can be too descriptive about *what* skills are needed and *when*, and therefore fails to embrace a more natural, fluid, and long-term approach.

The acquisition and development of ILS are not exclusive to young people leaving care but are more urgent for them. The United Nations has set international standards for all young people relating to acquiring and developing ‘life skills’ and developed guidelines for the Alternative Care of Children [48], giving weight to the need for a smooth transition to independence for young people raised by the state. For young people exiting care, a sense of rushed independence contradicts the idea that ILS and knowledge acquisition is a process rather than an event. McGhee and Deeley [49] point to ‘emotional readiness, resilience, and ongoing relational support’ being critical in supporting successful transitions from care. ILS acquisition depends on the ‘constructive processing of information, impressions, encounters, and experiences’ [50], which takes place throughout life and is developed over time, across the life course, with practice and support.

### Looking at longitudinal studies to understand trajectories across the life course

Longitudinal studies can offer important evidence to better understand outcome trajectories. Some previous reviews included longitudinal studies, but no reviews solely focused on such research methods. Longitudinal studies are unique in building an understanding of social changes and can contribute to re-shaping policy and practice. Longitudinal studies focusing on the care-experience have already shown the need for comprehensive transition planning for better post-care outcomes [51]. Further longitudinal research about outcomes for young people exiting the care systems [42] across the diverse range of life domains will enhance a deeper and broader understanding of the impact of having care-experience.

The first longitudinal OOHC study in Australia was undertaken by Cashmore and Paxman [52], interviewing 47 care leavers who had left OOHC, as well as their case workers at four time points. The ‘Taking Care Education Project’ in the UK interviewed 80 participants at two time points, finding young people felt their care-experience impacted their education negatively, from which they ‘could not recover’ [53]. This study found that one in four young people left school before 15 years of age. In the UK, the ‘By Degrees Project’ conducted by Jackson, Ajayi [54] interviewed 129 participants between ages 16 and 21 years and found schooling stability, and having at least one encouraging adult, helped 6% of young people continue to higher education. In 2011, The ‘Young People in Public Care Pathways to Education in Europe’ (YIPPEE) [55] study continued the ‘By Degrees’ study methods. However, YIPPEE included five countries’ data, attempting to map educational participation, identify conditions that support educational achievement, and understand young people’s educational and future-thinking constructs and trajectories. In the USA, the ‘Midwest Study’ [31] interviewed participants from three states at five time points across five years and found that educational, employment, and housing outcomes were poorer when compared to non-care-experienced peers. Findings also highlight mostly better outcomes for young people who experienced extended care.

Adopting a lifelong perspective concerning the development of ILS and knowledge resists the conceptualisation of independence and the abrupt and sudden move into adulthood that comes with it. Therefore, exploring what helps or hinders the acquisition and development of ILS and knowledge during emerging adulthood is important if understood as a developmental milestone that encompasses ‘neither childhood nor full adulthood’ [49].

The acquisition of ILS and knowledge is a topic explored in various disciplines, such as the mental health, disability, and rehabilitation fields. However, to the best of our knowledge, no systematic review has specifically targeted longitudinal studies of ILS and OOHC. We predict that observing longitudinal studies will show better after-care outcomes if the acquisition of ILS is supported by people, systems, and programmes. This systematic review aims to explore this by identify studies that have evaluated ILS and knowledge over time and to build a body of evidence of how young people in OOHC develop ILS. The objectives are to: (1) identify longitudinal studies relating to ILS for those with care-experience, especially during the transition from care; (2) summarise and appraise research that highlights factors affecting the development and utilisation of ILS for care-experienced young people; and (3) map and analyse the findings to identify common findings and gaps in ILS research relating to young people exiting OOHC.

## Methods

The methodology and reporting of this systematic review are based on the ‘Preferred Reporting Items for Systematic Reviews and Meta-Analysis’ (PRISMA 2020) [56] statement and checklist, which enhances the essential and transparent reporting of this systematic review. This study registered a ‘Protocol’ with OSF Registries (doi: 10.17605/OSF.IO/MJ3ZX) on June 5^th^, 2022. The Protocol noted that this review would conduct meta-analysis, however, it was decided this would not be an appropriate analysis given the breadth of included studies across varying domains of life. The protocol stipulated that the searches would be conducted using four databases, which was increased to seven to enhance the search. The Protocol proposed utilising the Cochrane ‘Tool to Assess Risk of Bias in Cohort Studies’ [57] to measure the quality of included studies, however, the Qualsyst [58] quality assessment framework was used instead due to the variability in study designs.

Internationally, interventions and approaches vary, and their themes take on a different focus, thus making valid comparisons of each study challenging. This review included a broad range of care experiences, including kinship, residential, foster care, and reunification. The review also sought to understand how preparation and planning services and programmes work best for the cohort. Programmes and interventions are of interest as they have been paid particular attention in the research area already, exploring what helps achieve positive outcomes. Ultimately, this review is interested in how ILS acquisition is supported and the transition to adulthood. Therefore, ILS are contructed as a life skill learned from others (people, systems, programmes), and timely acquisition of ILS supports better outcomes (e.g., health, housing, financial security) after young people leave OOHC.

### Eligibility criteria

The inclusion criteria applied as part of the search strategy for this review stipulated that articles needed to include studies that: (1) adopted longitudinal methodology, including follow-up studies; (2) focussed on young people who experienced OOHC and/or studies where young people were preparing to, or who had left OOHC; and (3) assessed or reflected on ILS (or overlapping terms that refer to ILS, which could have included ILS programmes, skill development and acquisition), and the impact different experiences of care have on ‘independence’ and post-care outcomes. Original articles that were published in English and peer-reviewed were considered for inclusion in this review. Any systematic, scoping or other reviews, conference papers, study protocol papers, or unpublished papers, including dissertations, were excluded from this review.

### Information sources and search strategies

There is a broad range of research looking at the outcomes of young people leaving care available across many databases. This systematic review used the following databases: CINAHL, Embase, ProQuest, PsychINFO, PubMed, Scopus and Web of Science.

Free text terms were identified concerning the following three concepts: Longitudinal Studies, Out-of-Home Care, and Independent Living Skills. Final search terms were agreed upon between authors. S1 Table shows the free text terms and search strategies, databases searched, and the number of results. Literature searches were conducted on the 20^th^ July 2023.

### Selection process

Retrieved titles and abstracts were screened by two reviewers between September 2022 and July 2023. Reviewers identified studies through a ‘yes, no, maybe’ grading system concerning the three main inclusion criteria noted above. Studies that met all three criteria were included and studies that clearly did not meet at least one of the criteria were excluded. Those studies with uncertainty about inclusion for one or more of the criteria were discussed between reviewers to reach agreement on inclusion or exclusion. The two researchers reviewing the studies met twice to discuss any studies that may not have met the inclusion criteria. Both reviewers independently screened papers identified at full-text level to ensure agreement on inclusion in the review.

### Data collection, data items and synthesis of results

Detailed tables were compiled to extract appropriate and relevant data from the included studies. Two authors synthesised the included studies in a table mapped against the following study characteristic’s (S2 Table) headings: Author, year of publication, country; Aims, objectives; Population, sample details; Timeframe, number of waves; Attrition rate; and Study limitations (as reported by authors). Regarding the specific interventions, a table was developed to organise findings (S2 Table) and information within the studies against the following categories: Process and procedure; Instruments and approaches; and Outcomes and findings.

To support and structure the findings the included studies were originally mapped against the following nine ILS domains which were used in both ILS surveys and interviews as part of the ‘Navigating Through Life’ (NTL) [16] project (a current Australian longitudinal, mixed-methods transition from care study): Money; Housing; Education and Training; Employment; Health and Wellbeing; Daily Living Skills; Personal and Social Development; Legal Rights and Responsibilities; and Safety. A Factor Analysis (FA) was conducted, and the nine ILS domains were reduced to eight and their domain names adjusted to: Financial Management, Accessing Support, Managing Housing, Education Planning, Job Seeking, Health Risk Management, Domestic and Self-help Task, and Managing Relationships. This review maps the included studies against the adjusted domains.

### Methodological quality and risk of bias

Two reviewers assessed the extracted data relating to methodological quality [55] agreeing on the final 17 studies that met the inclusion criteria. The additional ten studies sourced from citation checks were rated for quality by both reviewers. The likelihood of bias was limited, given that neither reviewer had any affiliations or conflicts of interest with the included studies. This review adopted Qualsyst [58] as a scoring system to appraise and check for study quality [58]. The Qualsyst checklist can be utilised for both qualitative and quantitative research. The checklist covers sample size, sample description, analysis approach (which includes methods), and reporting of results. An overall quality score is then calculated as a percentage (total score divided by maximum possible score multiplied by 100). A study percentage score of <50% is deemed poor quality, 50–69% is fair quality, 70–79% is good quality, and a score of >80% is a strong quality rating. Two reviewers assessed and classified study quality independently to assess study validity, errors, and biases [58]. Disagreements were resolved with a third party in the event of no resolution.

## Results

### Study selection

In total, 819 studies were retrieved from the seven electronic databases (CINAHL: *n* = 307; Embase: *n* = 156; ProQuest: *n* = 116; *PsycINFO*: *n* = 126; PubMed: *n* = 18; Scopus: *n* = 52; Web of Science: *n* = 44). After removing duplicates (*n* = 305), the remaining 514 papers’ titles and abstracts were reviewed and screened for inclusion by two researchers against the three inclusion criteria (Longitudinal studies; OOHC; and ILS). A total of 469 studies were excluded at this stage as they did not meet the inclusion criteria, leaving 45 studies meeting inclusion criteria, which were assessed for final eligibility and inclusion at the full-text level.

**Fig 1:**
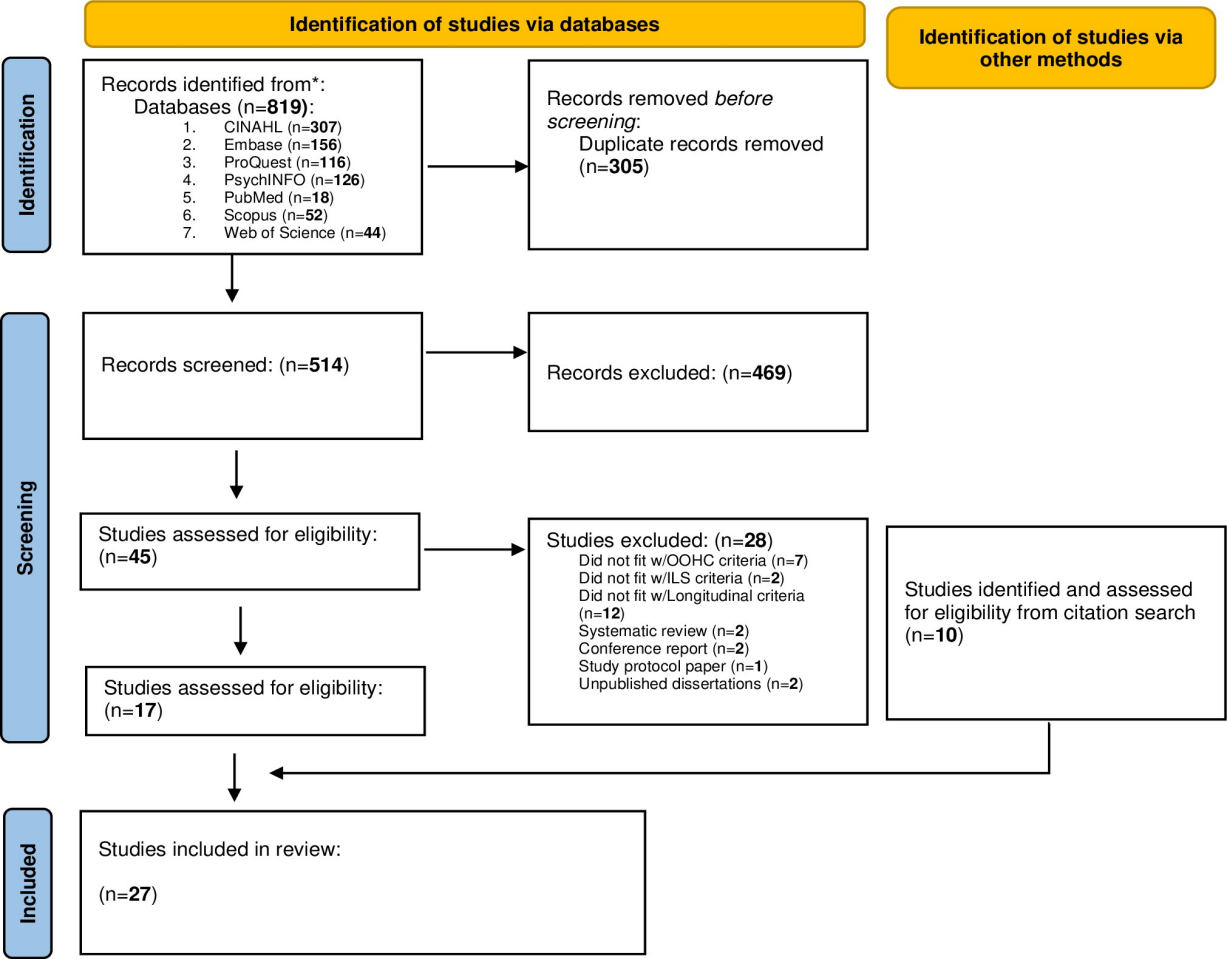
PRISMA flowchart.

Of the excluded studies, two were excluded as they were Systematic Reviews, two papers were excluded as they were conference reports, one paper was excluded as it was a protocol paper, and two papers were excluded as they were unpublished dissertations. A total of 17 studies were included in the data synthesis after meeting the final eligibility criteria. In addition to these 17, 10 other studies were identified by searching the reference lists of included articles and met the inclusion criteria. Fig 1 shows the PRISMA Flowchart [56] depicting the search and selection process.

### Characteristics and description of included studies

S2 Table summarises the study characteristics, including aims, objectives, and sample details from across the 2–4 waves of the longitudinal/follow-up studies, as well as attrition rates and any study limitations (as reported by authors). The studies were from a range of countries, with two-thirds of the studies from the USA (63% or 17 studies), followed by Israel (19% or 5 studies), with one study from each of the following locations: Canada, South Africa, Finland, Sweden and one cross-national study involving Norway, Denmark and England.

**Fig 2:**
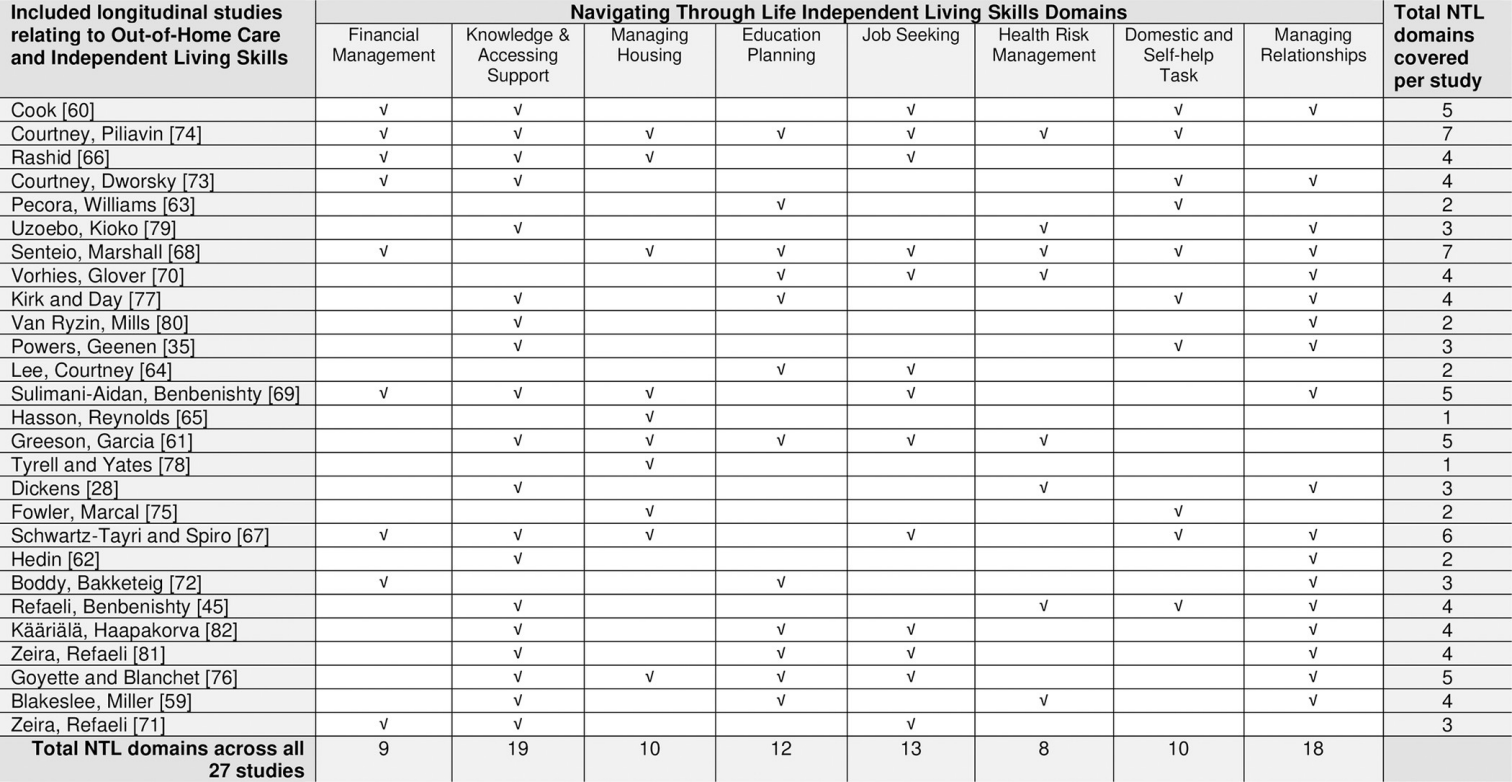
NTL ILS matrix.

All included studies met inclusion criteria relating to ILS; however, some did not clearly define ILS. A total of 14 studies defined ILS by how personal, practical, and relational factors help young people’s functioning and adjustment during the transition from care. The studies’ focus and outcomes covered different domains that impacted the acquisition or development of ILS. Fig 2 is a matrix mapping the studies against eight ILS domains (adopted from the NTL project [16]).

All the included studies reflected on outcomes for care-experienced people. Of the 27 studies, six [59–64] exclusively investigated experiences of foster care, eight [28, 65–71] included only participants who had been looked after in residential care settings, and the remaining thirteen [35, 45, 72–82] studies’ participants had a variety of different care experiences such as kinships, foster, and residential.

Of the included studies, eleven [45, 60, 63, 68, 70, 72, 76–78, 82, 83] adopted mixed-methodology, ten [35, 59, 62, 66, 67, 71, 73–75, 81] used qualitative approaches, and six [61, 64, 65, 69, 79, 80] used quantitative approaches. A total of five [35, 59, 61, 75, 82] studies conducted comparisons with general populations involving cross-national or control groups. Six [63, 72, 74–76, 82] of the included studies accessed administrative data for their analysis.

Cohort sizes contrasted, with a Min value of 5 [62] and a Max value of 2,913 [65]. Based on baseline sample size, the Median cohort size value across all of the 27 included studies equals 276. The age of participants across the included studies ranged from 16 years in the Hasson, Reynolds [65] study, to over 30 years of age in the Boddy, Bakketeig [72] and Pecora, Williams [63] studies. Consideration of how ILS are developed across the life course is reflected in six [59, 63, 65, 71, 72, 82] of the 27 included studies, which presented data relating to care-experienced people over 25 years of age.

The follow-up period varied, for example, the Hasson, Reynolds [65] study averaged 32.1 data collection points per young person, collecting housing data weekly. Whereas the Pecora, Williams [63] study reported on a follow-up over 30 years after the baseline data, exploring later life educational outcomes. Over two-thirds of the included studies (17 or 71%) reported on 2–3 waves across a 2–5-year period.

### Description of interventions

S3 Table presents a description of the interventions reported in each study, capturing details relating to the intervention process, the approaches and instruments used, and outcomes and findings from the intervention. Of the 27 included studies, twenty-three different interventions were described. The Hedin [62] study conducted a follow-up with some participants from a dissertation research project. The Refaeli, Benbenishty [45], Zeira, Refaeli [81], and Zeira, Refaeli [71] studies used the same data; however, they focused on different outcomes, one testing a model that predicts higher education aspiration [81], another testing a model to predict life satisfaction [45], and another conducting a ten-year follow-up [71].

Interventions, approaches and practice models varied, depending on countries’ legal responsibilities and, therefore, their respective focus on the cohort studied. All studies focused on ILS as an overarching theme for post-care outcomes. The included studies covered a range of primary themes covering specific needs, which would sit underneath the ILS umbrella, for example, mental health [59, 70], disability [35], housing [65, 67, 75, 76, 78], and education [63–65, 72, 76, 81, 82]. Over half (14/27) of the included studies focussed on individuals functioning, processing, and adjustment to life after care and provided a broad analysis which reflected on different ILS domains [45, 60–62, 64, 66, 68, 69, 71, 74, 79, 80, 82, 83].

Given the breadth of different ILS domains covered, comparisons cannot be made within this review. The differing themes and topics, interventions, and methodologies mean valid comparisons of the effectiveness of interventions cannot be undertaken. Across the included studies, there is no consensus or common notion of ILS used conceptually; thus, the focus is on understanding the ILS impact by mapping the studies against the eight NTL [16] ILS domains (Fig 2), which helps assess study content, support analysis, and identify gaps.

### Domains: Conceptual plotting of included studies

Fig 2 (‘NTL ILS matrix’) synthesises the findings of the included studies in this systematic review. The matrix includes the study and a tally against each of the eight ILS domains. The most common domains covered were Knowledge of Accessing Available Supports and Managing Relationships, represented in nineteen and eighteen studies, respectively. The least common domain used was Health Risk Management, addressed in eight of the included studies.

The matrix also represents an overall tally per domain. In summary, the fewest total of ILS domains covered was one domain in two of the studies [65, 78] compared with the most frequent being seven ILS domains in two studies [68, 74]. None of the studies covered all eight ILS domains.

ILS need to be considered across multiple domains of life [28]. The eight NTL domains provide a template describing the different areas that impact a young person’s life preceding, during and proceeding the transition from care. Research and practice should have ‘whole life’ [69] awareness, given that care-experienced young people are charged with navigating numerous areas of life, often creating a hasty move into independent living. Therefore, mapping results against the eight NTL ILS measures provides greater oversight.

### ILS domain 1: Financial management

Several financial factors were reported to impact ILS. Experience of financial instability was common, having a negative impact on outcomes and skill acquisition [67, 72]. Other negative examples included not having enough money to meet basic needs and often surviving on ‘low salaries’ with ‘low socioeconomic status’ [69]. In one study, fewer than half of the participants reported having bank accounts [73]. One study found four out of ten participants at both wave one and wave four (ten years after leaving care) were experiencing debt [71]. In another study, more than two-thirds were not ‘prepared’ to manage money when entering independent living [74]. This was also identified in 2012 [24] and 2020 [84], highlighting the perennial nature of the problem. Financial skills were observed to be learned in transitional housing projects [66, 67]. In four studies, links were made between education and employment success and economic security, stability, and financial capability [60, 68, 71, 73]; therefore, if young people achieve positive education and employment outcomes, they may be more likely to experience money and finance as less of a problem.

### ILS domain 2: Knowledge of accessing available support

The ‘Knowledge of Accessing Available Supports’ domain was the most frequently (19/27 times) explored across the included studies. Some studies had a particular focus on training across different ILS domains, which can enhance access to support when required, such as financial training [60, 66, 74], navigating community resources [79], and using mental health services [74]. The common goal across the included studies concluded that the transition from care should be less rushed and the impact of aging out of childhood and care should be extended. Studies considered how different transition points affect the acquisition of ILS, for example, changes in, or endings of, a placement, entering adulthood at age 18, changes of workers or services, or finishing a training programme. Schwartz-Tayri and Spiro [67] concluded that for young people who attend and complete training, the ‘leaving care crisis is postponed’ [67] or delayed.

Several studies highlighted that if young people develop individual personal growth characteristics such as having agency, taking responsibility, forward-planning, practising decision-making, enhancing self-concept, having a sense of purpose and empowerment, developing openness, self-determination, self-efficacy, self-esteem, and having aspirations, then they can be more likely to access support and services [35, 45, 59, 62, 76, 77, 80]. Work experience opportunities were found to build human capital [71]. In addition, having reliable and helpful relationships was noted as important in accessing support. As such, maintaining relationships with those involved in care, family relationships, mentors, or remaining connected to and being involved in provisions and services were deemed important [28, 59, 61, 67, 69, 73, 76, 81].

### ILS domain 3: Managing housing

Ten studies observed associations between housing and leaving care outcomes. Across the included studies rates of reported homelessness varied between 14% and 50% [73, 75, 76, 78]. Fowler, Marcal [75] found that exposure to specialist independent living services as well as extended foster care did not prevent homelessness. This may be the result of structural economic forces where there is greater demand than supply of housing. Although stable housing was found to promote the development of life skills [69], for example, Rashid [66] found in their study that transitional living programs helped young people maintain employment, save money and learn new skills. Housing stability is clearly foundational to experiencing success in other ILS domains [67, 74, 82]. As such, for some care-experienced young people, their often-transient experiences in care appear to be replicated in post-care housing and accommodation experiences.

### ILS domain 4: Education planning

Twelve studies covered the ‘Education Planning’ domain and factors affecting the development of ILS. Findings included the reporting of bi-directional associations between housing stability and educational stability, whereas education instability and ‘drop-out’ lead to academic delays [63, 70, 74, 76]. Such experiences of instability in the education system are linked to difficulties in other ILS domains [81], for example, educational attainment was associated with less likelihood of being involved in criminal justice systems, particularly concerning males [64]. Positive educational outcomes were supported by factors such as relational support and buddying/mentoring [59, 76].

### ILS domain 5: Job seeking

Thirteen studies focussed on the ‘Job Seeking’ domain, of which four studies from Israel focused on mandatory military service and, therefore, explored employment outcomes by default [67, 69, 71, 81]. One of these studies [71] observed that steady employment was more likely over time (up to 10 years post-care). Whereas employment instability and fragmented experiences of employment were observed in two of the other included studies [60, 82]. Other studies generally reported that young people had some experience of employment [68], which was supported by factors such as a focus on employability skills [66, 70], a presence of social support [81, 85] and positive housing experiences [74, 76].

### ILS domain 6: Health risk management

Eight studies mapped against the ‘Health Risk Management’ domain, of which one study noted nearly half (47%) of participants received support from a mental health service [74]. Across the studies, self-sufficiency [59, 68], resilience [28], life satisfaction [45], behavioural health symptomology [61], human development training [79], and self-determination [59] were noted as key themes that contributed to health and wellbeing for young people during the transition from care.

### ILS domain 7: Domestic and self-help tasks

Ten studies in this review addressed the ‘Domestic and Self-help Tasks’ domain. One study explored what was described as ‘life skills deficits’ [73] and the challenges this can create across ILS domains. The Courtney, Piliavin [74] study found that 76% of participants reported they received some form of daily ILS training, with 39% of participants reporting foster parents providing informal help to develop ILS and 32% learning ILS from formal Independent Living Programmes (ILPs). Cook [60] stressed that there is a need for young people to be taught concrete skills relating to specific aspects of life, such as job training and employability skill, and Kirk and Day [77] identified rational skills, such as taking responsibility for one’s own actions, forward planning, decision-making, and time management as key. Formal relationships, such as with professionals within the care system, and informal relationships, such as family and friends outside of the care system can help develop and acquire daily living skills [35, 45, 75].

One study reported that young people feeling unprepared was a major issue, finding that between one-third and a quarter of participants felt unprepared across many skill areas [74]. Therefore, continued opportunity to test, trial and practice ILS increases confidence and self-efficacy, which is a factor that contributes to an increased ability to handle independent living [45, 68, 69, 77]. Special independent living training and programmes were seen as an essential source of learning [67] and an important factor in increasing the likelihood of educational completion and greater socialisation [63, 85]. Although, the Fowler, Marcal [75] study observed a shortage of evidence regarding the effectiveness of ILPs.

### ILS domain 8: Managing relationships

The second most frequently researched domain in highlighting factors affecting the development and utilisation of ILS was ‘Managing Relationships’, with two-thirds of the studies (18/27) reporting on this domain. Better ‘independence’ outcomes emerge if young people have greater social support [73] and a personal sense of coping, which Zeira, Refaeli [81] describe as ‘personal resources’. Care leavers may have the skills to navigate the transition from care, but may not have the network of supportive relationships to optimise their ILS, which was described as having ‘limited relational capital’ [76]. Extended care or ‘after-care’ was identified as a support for better post-care trajectories [82]. Sulimani-Aidan, Benbenishty [69] identified social capital as a beneficial factor for ILS outcomes. One study found ‘socialisation’ (defined as decision making, offering opinion, expressing feelings and goal setting) as an important skill in achieving success in other life ILS domains [60].

Striving for independence is intertwined with a need for interdependence during early adulthood and can be built upon from good informal and formal relationships that are based on trust [62, 72]. Being empowered to achieve personal transition goals [35, 70, 77, 80] and developing a sense of agency through self-efficacy [45] and self-determination [59] were found to be positive contributors to independent living outcomes. The precariousness of outcomes for care leavers stems from not being able to acquire ILS through making mistakes and ‘trying again’ [72]. Therefore, it is essential to have opportunities to test out new skills and knowledge, which should start before leaving care [28], or when taking part in special ILS training programmes [67], and can contribute to what Uzoebo, Kioko [79] describe as ‘human development’.

### Methodological quality

No studies were excluded from this review on the grounds of quality. The included studies’ methodological quality was rated using the Qualsyst checklist by Kmet, Lee [58]. Twenty-one studies included within this systematic review were rated as strong (77.8% of included studies), five scored good (18.5%), and one study scored fair (3.7%). Inter-rater agreement was 100% with the results of the quality appraisal are summarised in S4 Table.

### Risk of bias assessment

It is important to recognise and understand the variation of the included studies, especially concerning the data used (including sample sizes, changes in population over time, and the needs and vulnerabilities of the samples and their view of their own experiences). Complete data was not known in one study because the full complement of waves was not completed [73]. Three studies identified missing data [59, 65, 80], which was seen as a potential for bias. It is more common in longitudinal studies that there can be missing data [86]. Two of the studies [76, 79] did not report on possible bias. Attrition, which relates to missing data for reasons such as loss of contact with a participant or participant withdrawal from a study, is particularly pertinent to capturing and presenting accurate data relating to outcomes for young people who have left care. As a result, interpretations and understandings could be distorted relating to challenges and issues experienced, similarly when considering positive outcomes. Attrition rates were reported in all but six studies [66, 68, 72, 75, 78, 79] (25%). Where attrition was identified, the lowest rate was 5.1% [45, 81], both from Israel and utilising the same longitudinal data. The highest attrition rate was 71% [62] which followed-up with a specific sub-section of the original cohort.

Validity could be questioned when participants in a sample self-identify issues: eight studies [65, 69, 70, 73, 75, 77, 79, 83] are based on self-reports. However, given the nature of the topic, capturing personal perspectives and interpretations of experiences provides invaluable insights. Similarly, reliability could be queried with small sample sizes where less powerful population estimates could be drawn. In this review, sample size was identified as small in seven studies [28, 35, 59, 62, 66, 68, 70]. The odds ratio was considered small in one study [63], which can create misleading results.

No control or comparison group was identified in four studies [35, 67, 68, 70]. A convenience sample was identified in one study, which could limit validity. Selection bias was reported in two studies [60, 73], which can happen if a group studied is not indicative of a whole population as there are ‘systematic differences’ [86], such as ‘unique characteristics’ [86] between participants and non-participants. Selection bias can be mitigated or reduced if participants are randomised, for example, into treatment and control groups, which eight studies did [28, 65, 69, 70, 74, 75, 77, 79].

Data in one study [60] was considered old (13 years) compared to one study [65], noting that follow-up points could have been too soon. Experimenter bias [70] was considered possible in one study, and omitted variable bias [61] was referenced in one study where relevant variables were excluded, both of which could highlight research control over the data and outcome. Additionally, there may be potential for ‘invested interest bias’ [87], given that many authors have previously researched the topic area.

## Discussion

This systematic review set out to identify, summarise and synthesise the development of ILS of young people transitioning from OOHC as reported in international longitudinal studies. The review’s focus on longitudinal studies was aimed at understanding how outcomes are impacted by what is known about young people’s ILS over time.

There is a ‘lack of reliable and comprehensive data on the characteristics, experiences, and transition pathways of young people leaving care’ [16]; therefore, this review has highlighted factors that promote the positive acquisition of ILS for care leavers, such as preparation at the right time for the individual, post-care support systems, recognition of the overlapping ILS domains, as well as a need for natural, fluid and long-term approaches to the development of ILS, across the life course.

ILS incorporate the overarching skills and abilities to live an independent life in adulthood [88]. In developing and practising ILS across different ILS domains, young people during the transition from care could benefit from a more positive adjustment to early adulthood. The 27 studies identified in this review, from across a twenty-nine-year period (1994 and 2022), explored 8 ILS domains. They built an understanding of young people preparing to, and having left care, as a diverse population with different experiences across their respective lives and respective countries. The systematic review found that there were no common definitions or measures of ILS used with this population to support comparative evidence in research. However, ILS acquisition may lead to independence by first focussing on interdependence (relationships, accessing supports and resources).The findings of this review are synthesised in a framework that consists of 8 ILS domains that contribute to ILS [16], which can support how researchers conceptualised ILS and what interconnected variables influence outcomes for young people leaving care. In this way, these domains underpin the construct of ILS.

A key aim of this review was to summarise factors affecting the acquisition of ILS and to identify gaps in the research when mapped against the 8 ILS domains. This review highlights that understanding ILS as a process of knowledge and skill acquisition can provide a greater understanding of the transition from care to post-care outcomes. The different ILS domains reflect what is required for active citizenship in transitioning to adulthood. ILS are learned over time and are context-specific depending on individual circumstances. ILS are acquired throughout life and will vary depending on external (social and economic structures) and internal (psychological, internal functioning) factors, which may help or hinder adjustment to independent life after care.

The USA was represented in nearly one-third (63%) of the included studies, which could indicate greater consideration of the importance of ILS given that independent living programs are mandated in the USA and in Israel conscription to military service is compulsory. Both countries may represent greater financial resources to conduct longitudinal research which also explains this finding, whereas in some Global South countries such resources may be less available. Combined, the USA and Israel [35, 45, 59–61, 63–71, 73–75, 77–81] dominated this review’s findings, indicating a need for a broader international understanding of how ILS are acquired longitudinally, building an understanding of the impact of different welfare systems, legal responsibilities and economic policies concerning care leaver ILS outcomes.

The individual ILS domains should not be examined in isolation nor out of context but understood as all-encompassing and holistic. Different ILS domains are interlinked and ultimately help or hinder each other. Sulimani-Aidan, Benbenishty [69] found difficulties in one ILS domain could undermine success in other ILS domains, which, in turn, impacts ILS acquisition and post-care life outcomes. Therefore, analysis of outcomes in one domain should also consider other domains. Conversely, success in one domain may positively affect another domain. It is possible that success could influence self-determination and motivation to build upon, or challenges in a domain can lead to entrenched challenges over time.

Themes mapped from each study measure practical and tangible outcomes as well as the reported experiences of young people. Fifteen studies (55.5%) evaluated four or more ILS domains, with most studies focusing on the ‘Knowledge of Accessing Available Support’ (19/27) ILS domain. The vulnerability of young people with care experience is well documented, especially at times of transition. This vulnerability may be explained, at least in part, by a lack of material and emotional support [14]. Support from trusted people optimises the conditions for developing ILS; however, this will vary amongst the diverse situations of young people. Some findings highlight that young people are not well prepared for the transition to independent living, nor are they provided with enough specialist support throughout the transitional time. The least represented life domain was ‘Health Risk Management’ (8/27). Twelve studies (44.4%) covered three domains or fewer, which indicates that research does not approach ILS holistically enough.

For young people to get the most out of the transition from care, they need to experience conditions that encourage practising ILS, which may be with specific people or taking part in ILPs. This will build familiarity and enhanced understanding, thereby developing confidence and mastery [89]. ILS development comes from acquiring new knowledge and practising that knowledge as opposed to the way policy and practice approach developing ILS, for example, by ticking off ILS from a checklist, rather than greater exploration at a personalised and individual level [74].

How young people develop knowledge and skills is an important consideration, as different meanings are attached to the process and outcome depending on the individual and their circumstances. ILS must not be viewed as a binary concept, whether one has a skill or not, or that a checklist adequately assesses gaps in knowledge and skills. ILS development is relational, and ‘trial and error’ is vital to create opportunities to determine how some young people may learn. For those not living in OOHC, this complex learning often occurs in their home environments under the guidance of family members. In contrast, similar opportunities may not be afforded to those in OOHC.

The variability of how care-experienced people respond to their unique, complex and, at times, uncertain experiences means policy and practice should not be linear and prescriptive if tailored, individualised services are a goal. Within this review, 14 of the included studies focused on young people’s adjustment to life after care. Concepts like ‘independence’ and ‘transition’ could be viewed as terms encouraging a short-term approach to ILS development. The exploration of longitudinal studies in this review embraces a longer-term view to ILS development, encouraging interdependency and recognition of a care continuum, particularly through early adulthood, where learning new skills and adapting to circumstances and situations is part of growing and developing throughout life. It is possible that transitioning from OOHC may be more difficult with a policy focus on ‘independence’, given that, for example, on an individual level, the concept of independence can leave young people feeling they are unsupported and under-prepared rather than autonomous.

Focus on independence is often linked to the build-up towards ‘aging out’ of care and the state’s responsibilities for these young people changing or ceasing, as is enshrined in legislation. It is not a ‘natural’ developmental milestone. Rather, it is a legally imposed responsibility of governments, social structures and systems of young people reaching the legal age of 18 years. This is regardless of their actual, physical, psychological, and developmental needs, the quality of their relationships, and what would be most beneficial for their futures. Transitions from care are often fragile due to temporary, ambiguous, and uncertain experiences before and during care. Care-experienced young people may have to reorientate, reconstruct, and reorganise how they operate during a key developmental stage, such as ‘emerging adulthood’ [42], compared to non-care-experienced counterparts.

There is a juxtaposition between independence and interdependence concerning young people transitioning out of care who are emotionally polarised between dependence and independence [9]. It is possible that transitioning out of care may be more difficult with a policy focus on ‘independence’. If there is a greater focus on healthy interdependency, transitional support may be more timely, tailored and comprehensive. Therefore, the international focus on independence may be counterintuitive and potentially detrimental to care leavers’ ILS acquisition and outcomes.

### Strength and limitations

A strength of this review is that the systematic search of the literature was comprehensive, searching seven databases to gather as many studies as possible to minimise the risk of appropriate studies being excluded. However, the included studies were only in English, potentially excluding relevant non-English studies that could provide rich findings concerning ILS acquisition.

The two reviewers worked independently to select the included papers for this review and score the studies’ quality, which enhances reliability and integrity. However, as with all study quality appraisals, there is a degree of subjectivity when evaluating the quality of studies.

A meta-analysis would have enhanced this study, but this was not possible given the heterogeneity of the studies in terms of their designs. Mapping the included studies to the 8 ILS domains was subjective, and future research may opt for different domain names, and also group identified studies differently.

This systematic review included 27 studies from ten countries and does not represent all welfare regimes. Children and young people services and systems lack a harmonised approach across countries [90]. However the authors hold in mind that each country represented in this review has their own unique context.Generalisability could be impacted by the different social contexts and accompanying policies and practices. For example, the USA dominates the studies included, where specific legislation means independent living programs are mandated.

### Recommendations for future policy, practice, and research

This review identifies different outcomes across ILS domains and synthesises barriers to success observed across studies. It also highlights that common policy terms, such as ‘independence’ and ‘successful transition to adulthood’, are mostly policy aspirations with little evidence about how these can best be achieved for all care-experienced young people. As a result, there is an opportunity to reconceptualise independence and take a more comprehensive, programmatic approach to transitioning into adulthood for young people leaving care. At the policy level, governments recognise the need for post-care supports. However, approaches to enact supports are not based on evidence about what contributes to better adult outcomes for care-experienced young people. Adulthood is fast-tracked for this group when their peers remain home for much longer. For young people living in OOHC, the legislative requirement to exit occurs when they are still cognitively developing. Most have experienced various types of trauma at home and during their care experience, and many are separated from family, living with and attempting to make sense of fractured relationships. Given there is no foreseeable decrease in the number of children and young people entering the care system. Therefore, more comprehensive pre- and post-care systems and structures are important for an increased number of young people transitioning from care.

Future research should include an analysis of ILS across a wider range of welfare regimes [91] to consider and compare cultural nuances and ILS priorities according to different countries. In Australia, for example, Indigenous critiques of the ILS may not be factored in some standardised practice, which may implicitly reflect a conception of ILS from a Western perspective. This requires particular attention given to Aboriginal and Torres Strait Islander over-representation in their care system. Findings in this systematic review represent only one country from the Global South (n = 1), where in India, for example, the concept of a ‘Sphere of Aftercare’ [92] was introduced to encourage policymakers and practitioners to understand the needs of young people transitioning from OOHC. This particularly relates to ILS acquisition, which was found to be low, especially among females, providing another example of societal and cultural differences that should be understood [93]. Within the Argentinian care system, most young people reported similar internationally understood feelings associated with the transition from OOHC, such as feeling insecure, afraid, and rushed into adulthood [94]. In Argentina, more children are in ‘institutional’ or residential care (over 80%) [94], compared to approximately one-third of young people in OOHC living in residential care settings in OECD countries [90]. This could mean more opportunities for young people in OOHC to develop ILS in economically advanced countries. Even in a comparative study where two countries’ welfare models are considered very similar, Norway and Sweden, Storø et al. [95], found differences in legislation and policy regarding aftercare support, but similarities in resources (such as the opportunity to access free higher education). Further research is needed to compare the range of models and approaches to ILS acquisition, as well as the diverse outcomes in Global South and Global North countries.

Future research should compare different countries’ OOHC policies by considering issues related to ethnicity, gender, poverty, academic aspirations and opportunities. This will highlight how resources impact opportunities for young people.

Considering context, ability, and relational capital could provide better outcomes when supporting effective ILS development. Future research could explore the implementation of the eight ILS domains as a framework for practice. Future research may also look at bidirectional impacts between different ILS domains, for example, inequalities and disadvantages relating to finance can impact how young people manage relationships. Also, further research exploring the intersection of different care experiences, personal characteristics or protected equality groups [96–98] and the impact on ILS development would provide a more comprehensive understanding.

A universal way of conceptualising or measuring ILS is needed. ILS should not be seen as interchangeable with independence. This could mean services risk approaching ILS acquisition as a short-term goal when it is a long-term and life-long developmental journey. The result may be that young people experience increased uncertainty and ambivalence about the *when*, *why* and *how* ILS are prioritised. An interdependency approach may reduce feelings of instant adulthood, isolation and loneliness, increasing wellbeing, resilience and actualisation, and ultimately leading to better ILS outcomes [99].

Building a greater understanding of the consequences of poor ILS and resulting care outcomes among professionals should encourage practitioners to prioritise ILS. In practice, individualised plans can mitigate instability and developmental disruptions and set goals for ILS preparation and practice. Furthermore, the process and experience of leaving care could be less challenging if positive relationships exist in the young person’s life [45, 100]. Without focusing on relationships, the misnomer of ‘independence’ and ILS could misguide some care leavers towards social and behavioural obstacles they cannot navigate. Nurturing interdependency first could provide foundations to move towards positive adjustment to life after care. Practitioners assessing interdependency may demonstrate a deeper understanding of ILS pre-, during-, and post-transition from care. The concepts of independence and interdependence should be further researched through studies with care-experienced adults who have long passed their transition-from-care stage to provide a reflexive understanding of their unique position and consider the life-long consequences of care in this context.

## Conclusion

Thousands of young people leave the care system in their respective countries each year when services cease or change across different ILS domains and a safety net is removed, leading to possible delays in moving on successfully through the transition from care and beyond. This review aimed to determine how young people who are leaving, and those who have left care, develop ILS through identifying and summarising longitudinal studies relating to ILS and young people transitioning from care. This critical stage of transitioning from care, where life beyond care can be fragile, is compounded by the vulnerability of care-experienced young people. To mitigate this, help-seeking behaviours should be developed with young people, and support systems and structures should be appropriate, accessible and focused, where ILS are highlighted through an interdependency lens. The discourse surrounding those in care and care leavers can sometimes emphasise independence when interdependence should, in fact, be prioritised, across different ILS domains. The services and people working with care experienced young people could provide time and space to learn ILS and to adapt to varying circumstances at an appropriate individualised pace, thereby enhancing transitioning from care and, ultimately, post-care outcomes via a focus on interdependence. Viewing ILS as an indicator of post-care success and understanding ILS as an essential overarching concept in leaving care practice could provide greater opportunities within services to build skills, knowledge, connections and confidence.

## Supporting information

S1 Checklist PRISMA 2020 for abstracts checklist. (DOCX)

S1 Table Database and search strategies. (DOCX)

S2 Table Study characteristics. (DOCX)

S3 Table Interventions studied. (DOCX)

S4 Table Methodological quality of included studies. (DOCX)
