# Supplementary material for: Understanding how young people transitioning from out-of-home care acquire and develop independent living skills and knowledge: A systematic review of longitudinal studies
Source: PLoS One. 2024 Jun 11;19(6):e0304965. doi: 10.1371/journal.pone.0304965 (PMC11166282; doi:10.1371/journal.pone.0304965)
Supplement: S1 Table — (DOCX) [file pone.0304965.s002.docx]

Fig 1: Database and search strategies

| **Database and search strategies** | **Limits** |
| --- | --- |
| **CINAHL:** (cohort stud* or longitudinal stud* or panel stud* or repeated measure* or prospective stud* or follow up stud* or follow-up stud*) AND (out-of-home care or out of home care or out-of-home placement* or out of home placement* or foster care or foster home* or foster youth or child*-in care or child* in care or young people in care or young people-in care or adolescent* in care or adolescent*-in care or children* home* or kinship or group home* or adopted child* or relative care or home-based care or home based care or orphan* or state care or public care or looked after child* or looked-after child* or substitute care or childcare system or child protection or child welfare or care leav* or care-leav* or leaving care or leaving-care or left care or transition* care or transition* from care) AND (independen* living OR independen*-living OR interdepend* OR inter-depend* OR self care OR self-care OR self determination OR self-determination OR coping OR skill acquisition* OR skill-acquisition* OR indepeden* life skills) | Title or Abstract |
| **Embase:** *As per Cinahl* | None |
| **ProQuest:** *As per Cinahl* | Title or Abstract |
| **PsycINFO:** *As per Cinahl* | None |
| **PubMed:** As per Cinahl | Title or Abstract |
| **Scopus:** *As per Cinahl* | Title or Abstract |
| **Web of Science:** *As per Cinahl* | None |
