# Supplementary material for: Understanding how young people transitioning from out-of-home care acquire and develop independent living skills and knowledge: A systematic review of longitudinal studies
Source: PLoS One. 2024 Jun 11;19(6):e0304965. doi: 10.1371/journal.pone.0304965 (PMC11166282; doi:10.1371/journal.pone.0304965)
Supplement: S2 Table — (DOCX) [file pone.0304965.s003.docx]

Fig. 3: Study characteristics

|  | **Author/ Year of publication/Country** | **Aims/Objectives** | **Population/Sample details** | **Timeframe/Number of waves** | **Attrition rate** | **Study Limitations (as reported by authors)** |
| --- | --- | --- | --- | --- | --- | --- |
| **1** | Cook [60]  USA | Presents findings from Phase two of the WESTAT longitudinal study to describe self-sufficiency outcomes of young people who left an ILS training scheme. | Phase I: 1644  57% female, 61% white, 47% disability (‘emotional disturbance’ was a high proportion).  Phase II: 810  Age range between 18 – 24 years when interviewed. | Phase I:  Records reviewed between Jan 1987 and July 1988.  Phase II:  Phone interviews conducted between Nov 1990 and Mar 1991 – 2 ½ - 4 years after leaving care. | 50% interviewed from the case record cohort. | The data was 13 years old at the point of study write up.  Possible selection bias given those selected for interview versus those not selected. |
| **2** | Courtney, Piliavin [74]  USA | To present early descriptive findings from the Foster Youth Transitions to Adulthood study (FYTA). | 149 met inclusion criteria,  141 interviewed at wave 1 & 113 at wave 2  (wave 3 not completed at time of publication). | 3 waves over 3 years (between 1995 and 1998).  Wave 2 = 12/18 month follow-up & Wave 3 approx. 3 years after leaving care. | 19.86% between Wave 1 and 2. | At the point of these findings being presented the study was not completed meaning the complete value of the data was not known (when multivariate statistical analysis takes place). |
| **3** | Rashid [66]  USA | Assessing outcomes of young people living in transitional living programs in Northern California, and comparing outcomes of young people who took part in employment training programmes (*n*=13) and those who did not (*n*=10). | 23 participants aged 18 – 22.  Sample recruited from Larkin Street Youth Services & the agency’s transitional living program, Avenues to Independence (ATI). | Status compared at intake, ‘discharge’ and 6 months post discharge. | Zero | Small non-random sample which can affect generalizability.  Specific care experiences were unknown. |
| **4** | Courtney, Dworsky [73]  USA | This was the first of a series of Midwest studies capturing experiences of young people age 19 and approximately 22 months after leaving care. | 767 = All eligible participants from Wisconsin, Illinois, and Iowa.  736 (95.8%) = completed the baseline interview.  Then 603 (82%) young people age 19. | Baseline in 2002.  This is data from the follow-up in 2004 (the 2nd wave). | 18%. | Selection bias due to unknown attrition.  Findings may not be generalizable across USA. |
| **5** | Pecora, Williams [63]  USA | To understand education achievements & comparing how they differ from other populations and identify key factors linked to school completion when in foster care. | 1,082 interviews conducted from the 1,609 case records accessed.  Average age at time of interview was 30.5. | 2 waves (baseline and a follow-up interview after leaving care between 1966 and 1998). | 26.6% (total # of interviews conducted from total # of case records). | The odds ratios for the continuous variables are relatively small, despite predictors being statistically significant. |
| **6** | Uzoebo, Kioko [79]  USA | To examine the perceived life skills needed to become successful members of the community, for young people preparing to leave and having left care. | 89 completed assessment and questionnaires.  16 out of a possible 24 invited and agreed to participate in the focus groups. Average age was 16.  Majority of participants are defined as having ‘special needs’ with a range of psychiatric issues. | Follow-up = 18 months after intake. | Zero | Not reported but possible selection bias. |
| **7** | Senteio, Marshall [68]  USA | To determine whether the TRAC programme provides the opportunity for a stable life in adulthood. | 24 participants from the housing assistance programme.  Mean age 20.3.  54% female.  54% African American. | Screening data collected at two points, at least 1 year apart. | Zero | No comparison group.  Small sample.  Timing of data collection. |
| **8** | Vorhies, Glover [70]  USA | Aims to understand the effectiveness of a residential programme for pregnant and parenting young people with severe mental illness. | 25 females, aged 18–21 years old. | Monthly status changes were tracked as a measure of programme effectiveness.  ‘Time 1’ = assessment within a few weeks of intake; and ‘Time 2’ = assessment approx.. 10 months later. | 28% (25 young people at ‘Time 1’ and then 18 at ‘Time 2’). | No control or comparison group.  Possible ‘experimenter bias’ as staff completed data collection.  Small sample. |
| **9** | Van Ryzin, Mills [80]  USA | Study examines young people’s understanding of transition from care via YET questionnaire. | 569 young people in the sample aged between 16 and 22.  540 participated by completing the YET measure at baseline and 216 at follow-up. | 2 waves = baseline  and 6 follow-up. | 60% | There was missing data – Little’s test completed which was not significant. |
| **10** | Kirk and Day [77]  USA | To evaluate young people’s self-report of a summer camp, study has a focus on education. | 38 eligible to participate.  34 young people took part. aged 15 – 19.  15 in 2008 and 19 in 2009. | One short-term follow-up took place 3 months after baseline with 23 young people. | 32% | Participant sample selection bias and completion of self-report questionnaires. |
| **11** | Powers, Geenen [35]  USA | Looks at whether transition outcomes are improved by comparing levels of self-determination. | 69 (33 in the intervention group and 36 in comparison group). | 3 waves over 2 years (baseline and then follow-up one year later). | 13% post-intervention and 11% at follow-up. | Sample size was small, which could raise bias in reporting. No control group. Mixed models were used in analysis - allowing to repeat measures. |
| **12** | Lee, Courtney [64]  USA | The study explores relationships between extending care past 18 and self-reported criminal and legal involvement. | 732 participants  (474 from Illinois, 195 from Wisconsin, and 63 from Iowa) | Four waves of data collected biannually: before they left care at 17, age 19, age 21, and age 23.  Baseline data collected between May 2002 and March 2003. | Wave 2 = 18% (82% of original sample).  Wave 3 = 19%  Wave 4 = 18% | Use of Audio Computer Aiden Self-Interviewing (ACASI) was seen to reduce desirability Bias.  Use of dichotomous variables may limit models.  This study does not differentiate between those with strong bonds to the foster care system and other institutions from those with weak bonds. |
| **13** | Sulimani-Aidan, Benbenishty [69]  Israel | Examines military service, economic status and stability, and satisfaction with accommodation 1 year after leaving care and looking at associations between outcomes and personal and social resources. | 277 then 236.  60.6% were male and mean age 19.5. | 2 waves (baseline and follow-up) over 1 year. | 14.80% | Study is based on the self-reports only, not others’ views. No examination of subgroups or different demographics leading to caution interpreting correlations found, as indicating causality. |
| **14** | Hasson, Reynolds [65]  USA | Explores gender differences re: education, employment, and housing experiences for young people in a transitional living programme. And how gender, education and employment are associated with the time to secure independent housing. | 2,913 out of a possible 3,551 (from across 5 states).  Young people enrolled on a transitional living program aged between 16 and 26. | Average of 31.2 data collection points per young person over a 4 year period (2010 - 2014). | 18% (removed from sample using listwise deletion of missing study covariates). | Timing of data collection is significant i.e. post housing crisis nationally following economic downturn. |
| **15** | Greeson, Garcia [61]  USA | To evaluate the effectiveness of the Life Skills Training (LST) Programme and exploring support differences for young people based on race and ethnicity. | 482 young people (234 from the LST program and 248 from the control group).  17 years old at recruitment. | Baseline – where 97 % of eligible young people were interviewed – and two follow-ups. | Follow-up 1 = 9% and follow-up 2 = 12%. | Varied approach by staff in delivering the training.  Omitted variable bias – given potential personal factors that provide support that may already be in place despite participation in LST. |
| **16** | Tyrell and Yates [78]  USA | The study documents changes in housing quality during the first 24 months following leaving care, adopting a risk and resilience framework. | 172.  66% female; Ave age = 19.63 years | 3 waves. | Not reported. | Non-random sample.  Data is retrospective. |
| **17** | Dickens [28]  South Africa | Determines the contribution of resilience to independent living one year after leaving the care of GBT in South Africa. The cohort left GBT residential care between 2012 and 2015 and were all aged 16+. | 69 young people initially (aged 16+), then 52 young people (who completed the one-year outcome interview in 2016). | 2 waves over a 3 year period (baseline and follow-up). | 25% | Measures rely on self-reporting. Study has a small sample, reducing statistical power. Risk of type 1 errors due to large number of statistical tests conducted (551). Results cannot be generalised as data is drawn from a single organisation. |
| **18** | Fowler, Marcal [75]  USA | Examines housing instability and homelessness and tests if young people aging out of care experience higher risk of homelessness. | 350 out of a possible 5873. | 3 waves (baseline and follow up at 18 and 36 months) over 3 years. | Zero | Follow-up points may be too soon to understand housing trajectories. Causal inferences are not applied given the observational design. Pre-existing differences that influence housing problems not considered. |
| **19** | Schwartz-Tayri and Spiro [67]  Israel | Establishes how young people are doing 4 years after leaving a transitional housing project. | 25 participants (age 21 - 27) out of the original 56 young people eligible. | 2 waves (baseline and then follow-up) over 5 years (2007 - 2012). | 65.40% | A comparison group could have found participants to be even worse off. |
| **20** | Hedin [62]  Sweden | Captures young people’s perspective and own understanding of their lived experiences. | 5 - all of whom were placed in kinship (family and friends care) care. | 2 waves (baseline and then follow-up). Original interview in 2008/2009 and then follow up in 2013. | 71% (17 participants at point of original interviews). | Small sample size = high risk that observations could be due to chance. |
| **21** | Boddy, Bakketeig [72]  Norway, Denmark, England | Considers how different economic contexts and how wider social supports jeopardise transitions from care outcomes. | 75 (Norway=24; Denmark=30; England=21). | 3 waves (interviews) over 1 year. | Zero | Study does not analyse young people who have experienced the most precarity as all participants are in education, employment or training. |
| **22** | Refaeli, Benbenishty [45]  Israel | Explores whether life satisfaction changes at one and four years after leaving care. | 276 at T1; 234 at T2; 222 at T3 (plus 16 narrative interviews at wave 3). Data is collected from young people across 26 residential care settings.  Average age at T1 was 18.96 and at T3 was 23. 55% of respondents lived with parents. For the qual. data there was a 50/50 gender split. | 3 waves over 4 years. | 15.2% between T1 & T2.  5.1% between T2 & T3.  19.6% between T1 & T3. | Those who did not take part at T3 may not be experiencing life satisfaction, so attrition rate may compromise the representativeness of the sample. Life satisfaction measure has not been used so comparisons cannot be made. |
| **23** | Kääriälä, Haapakorva [82]  Finland | Explores early adulthood education and employment trajectories and examines care history factors that predict the trajectory. | Used individual-level data from a 1987 longitudinal Finnish Birth Cohort study (Paananen & Gissler, 2011).  N = 59,476, of whom 1,893 were placed in care before turning age 18. | Data collection 1987 – follow-up data collection in 2012 (foetal stage until age 25).  1,893 individuals (3.2%) were placed in care before age 18 | Zero. | The dataset did not include information regarding children’s pre-care experiences and characteristics. |
| **24** | Zeira, Refaeli [81]  Israel | Testing a model that predicts Aspirations toward Higher Education (AtHE). | 276 at T1; 234 at T2; 222 at T3. Data is collected from young people across 26 residential care settings - 9% were already in higher ed  60% male. At T1 average age = 18.96 at T3 = 23.01. | 3 waves over 4 years. | 15.2% between T1 & T2.  5.1% between T2 & T3.  19.6% between T1 & T3. | The convenience nature of sampling limits validity of conclusion. |
| **25** | Goyette and Blanchet [76]  Canada | Focus on living conditions and  housing stability, education and career paths and personal difficulties in accessing social and health services in relation to 17 – 21 year olds transitioning from care. | Total pop. Size = 2,573.  Total w/contact details = 1,687.  Total completed 1^st^ questionnaire = 1,136. Total interviewed at wave 2 = 835.  Average age at wave 1 = 17.2 and at wave 2 = 19.07. | 2 out of 3 waves reported.  Interviews over 2 years (between April 2017 and Dec 2019). | Wave 1 = 44% of entire pop.; 67% of contact details pop.  Wave 2 from 1 = 36% | None reported. |
| **26** | Blakeslee, Miller [59]  USA | Testing the efficacy of the My Life [35] study ten years on.  Participants were enrolled at university and college for at least 2 years and self-identified as having mental health difficulties. | 35 of the 65 eligible young people recruited (aged 18 – 26).  The 35 were a small pilot RCT subgroup - from both intervention group and comparison group. | 3 waves over 4 years (2015 - 2019). | 46.2% of recruited young people. | Small sample size impacts the ability to randomise.  Missing data from 12 participants (34%).  Sample includes students with self-identified MH problem rather than official diagnosis. |
| **27** | Zeira, Refaeli [71]  Israel | Examines employment and economic self-sufficiency outcomes (including self-reports) over time, specifically, from 1 year after ageing out of care (about age 19) to 10 years later (about age 29). | 276/285 approached and originally recruited from educational and welfare residential settings (around a 30/70 split). Seven youth villages were selected for the convenience of their geographical location. In these settings, we approached all youths who were in school on the day of the survey.  Of the original sample (at T1) 84.1% had worked at some point. | 4 waves: T1 = 276/285 who were just about to leave care.  T2 = a year later 235 participated.  T3 = 3 years later 222 young people were interviewed.  T4 = 10 years post-care 151 participants. | T2 = 85.1% of original sample.  T3 = 80.4% of the original sample.  T4 = 54.7% of the original sample. | There was a lower response rate at T4.  The study is about subjective and personal perceptions. |
