# Supplementary material for: Understanding how young people transitioning from out-of-home care acquire and develop independent living skills and knowledge: A systematic review of longitudinal studies
Source: PLoS One. 2024 Jun 11;19(6):e0304965. doi: 10.1371/journal.pone.0304965 (PMC11166282; doi:10.1371/journal.pone.0304965)
Supplement: S3 Table — (DOCX) [file pone.0304965.s004.docx]

Fig 5: Interventions studied

|  | **Author/ Year of publication/Country** | **Process and procedure** | **Instruments and approaches** | **Outcomes and findings** |
| --- | --- | --- | --- | --- |
| **1** | Cook [60]  USA | Reviewing a national sample of case records from the **Westat Inc. study (National Evaluation of the Title IV-E Foster Care Independent Living Program for Youth)**.  A multistage, stratified design with probability sampling – at the 3 stages of selection: state, county clusters, and young people 16 and older.  Phone interviews conducted at Phase 2 (2.5 – 4 years after leaving care). | Cases were weighted up to represent a much larger sample of young people.  Skills were assessed by 23 questions at interview. Employment status, economic self-sufficiency, parenthood, support network, drug and alcohol use, housing, and health care were explored.  Outcomes compared to other study results and the general population. | Young people who received independent skills training did better than those who did not on 8 outcome measures.  Training particularly supported money-management, education, employment, health care, life satisfaction, and not being a cost to the community.  Poor employment stability, low education status and pregnancy rates were precursors for future instability.  Young people who were in care had comparable outcomes to those living below the poverty line. |
| **2** | Courtney, Piliavin [74]  USA | **The Foster Youth Transitions to Adulthood study (FYTA)** utilised descriptive data and young people narratives to clarify how Government services help transition from care to independence.  The study used data from Wisconsin Human Services Reporting System to identify participants who were aged 17 and 18 years old and who had been in care for at least 18 months and who came from across 42 different counties. | During interviews all aspects of life were discussed with some self-report surveys completed via The Multidimensional Scale of Perceived Social Support (MSPSS) and Wide Range Achievement Test of Reading (WRAT-R). | Experiences of participants suggest child welfare systems might be able to improve the passage to independent living by building on family strengths, minimizing negative family impact, and providing a safety net and concrete assistance in housing and employment.  Greatest support self-identified was from ‘significant others’ followed by friends, foster family and then family. This was consistent across wave 1 and 2.  One-quarter to one-third reported a lack of preparedness for independence. |
| **3** | Rashid [66]  USA | Young people were recruited via the **Larkin Steet Youth Service Avenues to Independence program** – a transitional living programme which include daily living skills, employment training, education, and supervised practice living. | Young people are referred via homelessness, mental health and other youth services.  The aim is to replicate ‘real-world’ experiences and consequences.  Young people, as residents, are also required to work towards general educational development (GED). | Transitional living programmes can provide opportunity to find and maintain employment, save a substantial amount of money, learn daily living skills, experience a “mock” real-world living situation, and achieve an hourly wage necessary to sustain independent living. |
| **4** | Courtney, Dworsky [73]  USA | **MidWest Study** focussed on young people leaving care across three states  Interviews conducted at each wave with young people still in the care of the state and this paper represents findings from interviews at wave 1 and 2. | Cross-cutting topics in interviews include: education, employment, housing, justice system involvement and physical and mental health. | Wave 1 found young people were more likely to be held back a year, twice as likely to be suspended, four times more likely to be expelled and more likely to be involved with the criminal justice system compared with the general population.  Wave 2 found young people would benefit from continued support from services beyond age 18, given the multiple challenges faced.  Most participants did not fare well compared to general populations across various life domains, but those experiencing extended care had better outcomes. |
| **5** | Pecora, Williams [63]  USA | Educational outcomes were compared between **The Casey Foster Care Program** ‘alumni’ and other populations.  To be included in the study participants needed to have lived with a Casey fostering family for 12 months or more and had aged out of care at least 12 months prior.  All participants were in foster care as part of the Case Family Program between 1966 and 1998. | Data was obtained from case records, where interviewees were identified from foster care case record analysis.  Study interviews were from across 13 different states. | High school completion rate at follow-up was higher than the general population (86.1% compared to 72.5%) – suggesting educational achievement delay for those with care-experience.  Achievement of BA degree was lower than gen. pop. at 10.8% compared to 24.4%.  High school completion predictors included: entering care at an older age, fewer placement changes, employment exp. while in care, independent living training while in care, and less criminal behaviours. |
| **6** | Uzoebo, Kioko [79]  USA | Intake and follow-up assessments conducted within the **VISIONS** **program** – a community-based human development programme for young people in care. | Assessment of money management, daily living skills, accessing community support, work, study habits, and communication.  Ansell Casey Like Skills self-report used for quantitative data collection.  Life Skills Evaluation Questionnaire and focus group discussions used for qualitative data collection. | Self-report showed higher mastery of skills in daily living skills, work life, money management, and self-care. Participants reported a 52-55% increase in skill acquisition.  Mentorship supported skill acquisition. Young people struggled to balance abstract independent living skills constructs with practical or hands-on opportunities. |
| **7** | Senteio, Marshall [68]  USA | **Transition Resource Action Centre (TRAC)** – Participants recruited via a residential transition service where indirectly supervised apartment are provided. | The Self-sufficiency matrix is used as a self-assessment tool and a management tool by TRAC. | TRAC was an effective programme for homelessness reduction with participants accessing transitional or temporary housing.  Unemployment was found to decrease. Income, accessing health care, life skills, support networks, and community involvement all increased – potentially contributing to greater housing stability. |
| **8** | Vorhies, Glover [70]  USA | Participants recruited from the **Thresholds Mothers' Project (TMP)** – A programme providing young mothers with the skills to live independently and to care for themselves and their children. | Self-reports include: Brief Symptom Inventory self-report; Child Abuse Potential Inventory self-report; Parent Opinion Questionnaire; Parenting Stress Index.  Self-report scores are compared bi-annually by staff assessments, which include: standardized assessments of parenting competency and stress; child maltreatment risk; and mental health symptoms. | Programme participation is associated with positive changes in participants' familial relationships, family responsibility and care, proper parenting behaviour and feelings, and parental distress and competency, but no change in mental health symptoms.  Programme participation saw changes in education and employment, and low numbers of suspected and substantiated child maltreatment reports. Negative behaviours observed were increases in frequency of AWOL incidents and subsequent pregnancies. |
| **9** | Van Ryzin, Mills [80]  USA | **Andrus Family Fund** initiated the project where the Youth Experience of Transition (YET) questionnaire was completed to assess perceived preparation for the transition from care.  Young people completed surveys and a transition coach was employed as a support. | Youth-serving agencies and organizations in this project integrated the Transitions Framework into their programming and services in order help young people improve their skills in managing the difficult changes they face.  Exploratory factor analysis adopted at baseline and confirmatory factor analysis at follow-up. | Young people became more comfortable and more open and reflective regarding the transition.  They were better equipped to handle the change, resulting in lower levels of internalizing symptoms. |
| **10** | Kirk and Day [77]  USA | **The Michigan Educational Opportunities for Youth in Care (MEOYIC)** preparation for leaving care program is a residential camp on Michigan State University campus.  Young people were recruited via their involvement with the child welfare system in Michigan.  A mixed method short-term longitudinal evaluation design used to assess outcomes related to Independent Living Program (ILP) content, self-efficacy, skills, attitudes and program quality. | Evaluation of first 2 years of the short-term 3-day residential campus-based summer ILP.  Outcome evaluation by pre-post questionnaires and Michigan Educational Opportunities for Youth in Care Questionnaire (MEOYICQ), with semi-structured interviews with ILP coordinators and focus groups with youth to evaluate process. | Youth reported an increase in personal, educational and life skills development outcomes upon completion of ILP.  However, slight decrease on most outcomes reported at follow-up (3 months post-ILP).  ILP leadership by foster care alumni and role modelling was seen as the highlight of the program.  Enhanced sense of self and increased responsibility taking were reported. |
| **11** | Powers, Geenen [35]  USA | The study explored outcomes of exposure to the **TAKE CHARGE** model for young people leaving care who have disabilities. As well as comparing outcomes with young people who receive standard independent living services.  Young people were identified via the state list, which was cross referenced with special education records.  Participants recruited needed to be receiving special educational services; be aged between 16.5 - 17.5, be in care for at least 90 days, and attending a large school district in the target area.  Young people in the TAKE CHARGE intervention group participated for 12 months – this included weekly coaching sessions and quarterly mentoring workshops. Young people were supported to practice and develop skills and provided with a self-help guide. | The study used experimental and longitudinal methodologies.  The model = 2 independent groups (control and comparison) x three repeated measures (baseline, post-intervention and one year follow-up) design.  Self-determination coaching and mentoring for adults with disabilities was adopted.  The ARC Self-determination Scale, The Quality of Life Questionnaire, Transition Planning Assessment, and an Outcome Survey were all adopted and tracked over the three data collection points. | Evidence that self-determination enhancement is effective in supporting youth in foster care and special education to promote their transition status.  Self-determination enhancement is statistically documented as a partial mediator. |
| **12** | Lee, Courtney [64]  USA | Uses data from the **Midwest Study**.  Participants had to be in care for at least one year.  The study collected self-reported information across different domains. | Survey’s were administered by Audio Computer Aiden Self-Interviewing (ACASI).  Seven self-reported criminal outcomes were used. | Employment and race are associated with criminal behaviours but not legal system involvement, and care status and age are associated with legal system involvement but not criminal behaviours.  Among men, educational attainment and employment were associated with lower odds of both legal system involvement and criminal behaviours, but not for women. |
| **13** | Sulimani-Aidan, Benbenishty [69]  Israel | The study included data from an Israeli longitudinal study examining the status of care leavers 1 year after leaving care in core areas (their military service, economic status and stability, and satisfaction with their accommodation).  The population was recruited from across 26 residential settings.  A survey was completed just before leaving care and 1 year later. | At wave 1 young people completed the following self-report questionnaires: the Life Orientation Test-Revised, readiness for independent living questionnaire, Relationship with Father/Mother Questionnaire, Medical Outcomes Study (MOS).  As well as questions relating to military adjustment and accommodation satisfaction and economic status. | Social capital can be gained from military service.  Perceived readiness to leave care predicted better economic status, which went hand in hand with better ILS.  Biological relationships also developed ILS.  A clear need for practical preparation while in care was identified. |
| **14** | Hasson, Reynolds [65]  USA | Considered housing experiences for young people in a **transitional housing program (THP)** and differences in gender in relation to education and employment. As well as understanding how gender, education and employment are associated with the time it takes to reach ‘secure independent housing’ status.  Data collection points were on average every 7 days, at points when young people met with a specialist while enrolled in the program. | Housing security was measured using the following categories: ‘secures independent’, ‘secure dependent’, ‘semi-secure’, and ‘insecure’, which was self-reported by young people at their weekly meeting with the project specialist. | A unique set of data given the number of data collection points.  Results show positive relationships between education and housing outcomes.  Over time a greater number of women experience more ‘secure housing’ and associations identified between secure housing and education attainment and employment.  Initial success was observed in the evaluation of this THP. |
| **15** | Greeson, Garcia [61]  USA | **Life Skills Training (LST)** classroom-based programme aimed at preparing young people to leave care.  Study uses secondary data form the randomized controlled trial: **Multi-Site Evaluation of Foster Youth Programs.** | Surveys conducted to understand prosocial activities, educational involvement, living arrangements, employment, victimization experiences, placement instability and behavioural health. | No racial/ethnic difference in the social support trajectory was detected.  Classroom-based learning was identified to provide informational support but not tangible and emotional support that relationships provide. |
| **16** | Tyrell and Yates [78]  USA | Young people were part of the **Adapting to Aging Out** study.  Data collected via face-to-face interview to measure housing quality month-by-month. | Multilevel model analysis of Sociodemographic variables, Childhood maltreatment variables, and Out-of-home placement variables conducted to identify specific risk and protective factors that accounted for housing quality. | Significantly low housing quality for 10% 2 years after leaving care. But 40% experience gains in housing quality.  Parenting status and education level were associated with better housing quality. |
| **17** | Dickens [28]  South Africa | The **Growth Beyond the Town (GBT)** programme (one of the largest therapeutic residential child and youth centres in South Africa) population help understand the contribution of resilience to independent living one year after leaving care. | Young people completed the Youth Ecological Resilience Scale (YERS) at the point of ‘disengagement’ (leaving) from care, which uses a 5-point Likert scale.  At the follow-up point participants completed a structured interview as well as self-administered scaling. | Development of a social-ecological resilience framework for care-leaving reflecting on interactional and environmental factors. |
| **18** | Fowler, Marcal [75]  USA | Using **The National Survey of Child and Adolescent Well-Being** to examine housing instability and homelessness. Data for this study was accessed from the NSCAW II (second cohort).  Comparisons across three groups (to test risk of homelessness): (i) reunified young people; (ii) young people who age out of care and receive Independent Living Services; and (iii) young people in extended care.  Adult caregivers and children aged 11 and older were interviewed via computer-assisted technology.  Housing issues were captured at 18 and 36 months. | A two-stage stratified sampling design was adopted. First, dividing the US in to 9 sampling strata and second, randomly selecting families from a monthly list generated by each strata.  Self-reports were captured relating to whether young people had experienced independent living services. Youth demographics were captured as well as county characteristics (both of which included population based data – census). Linked data was incorporated to understand extended care status.  Measures included: Housing problems (measured by structured interviews) – questions were adopted from the National Longitudinal Survey of Youth (NLSY97); and aged out status was explored – specifically by different child welfare experiences/status. | Results suggest young people leaving care exhibit similar probability of literal homelessness as adolescents who were never in care.  Reunification is associated with lowest probability of homelessness.  Independent living services and extended care are not associated with reduced risk of housing problems.  Findings overall highlight the vulnerability of child-welfare young people re: housing problems. |
| **19** | Schwartz-Tayri and Spiro [67]  Israel | The study looked at how participants in the **Bridge to Independence program** (a Transitional Housing Project) are doing 4 years after the programme ended, with a specific focus on military and civilian service, housing, tertiary education, employment income, health, leisure activities, social relations, and satisfaction with life. Almost all participants aged out of residential homes and participants already lived in apartments throughout Israel.  The sample is defined as a convenience sample (56 young people across 7 apartments between 2007 and 2012). The study also sought retrospective evaluation of experiences. | The study targeted young people in residential or foster care and those who do not have family or kin support.  A mostly multiple-choice questionnaire was completed. As well as semi-structured in-depth interviews were conducted. Interviews were conducted over the phone.  Personal bias checked by comparing participant characteristics of the 25 interviewed and the 31 not participating. | Findings highlight a need to maintain close contact with THP participants and also to strengthen the programme by preparing participants to live on their own.  Relationships with staff were seen as a real source of support - but after the programme is finished, turning to staff for support was seen as an admission of failure.  Struggles identified across life domains were linked - financial issues, not being able to continue studies, housing instability, leading to social isolation.  Transitional Housing Projects may postpone the leaving care struggle. |
| **20** | Hedin [62]  Sweden | Study looks to capture young people’s own understanding of their lived experiences.  17 participants in original study (2008-2009), a follow-up was conducted with 5 out 11 (who were contactable) young people.  In-depth interviews were conducted with the 5 participants, exploring perceptions of their everyday life. | Young people completed scaling relating to foster family functioning, additionally network mapping was conducted with young people.  Study has a qualitative interpretative design with experience sampling method (ESM) is adopted: using 'beepers' to capture 'here and now' situations with young people. | Having agency was a result of having a secure base. Young people’s agency can support overcoming challenges, build maturity and confidence.  Having the option to resume study later was of importance. |
| **21** | Boddy, Bakketeig [72]  Norway, Denmark, England | The study utilised the **Against All Odds?** research, where secondary analysis of administrative data is combined with longitudinal, and cross-national qualitative interviews and documentary comparing policy and practice.  Interview one used a ‘life chart’ to map experiences across a range of life domains. Participants then took photos for a week about what mattered to them. A week later the second interview was conducted focussing on the photo content. Approx. 12 months later the third interview was conducted. | Participants were recruited from non-governmental organisations, statutory services and social media. Participants self-identified as care leavers and as 'doing well'.  Wider social supports/frameworks and economic contexts are considered. Recognition that Norway, Denmark and England all differ in ‘after-care’ support, as well as early adulthood norms. | Concerns about finances and living situations were evident when comparing all three countries. Cross-national differences between England and the two Scandinavian countries is evident in welfare entitlements - which may get in the way of an ability to be flexible in approach and practice.  The research shows that pre-, during, and post-care experiences shape risk of precarity. This study is a reminder that the dependence of care leavers on formal systems arises because they cannot rely on informal family resources to scaffold them. |
| **22** | Refaeli, Benbenishty [45]  Israel | Explored whether life satisfaction changed at one and four years after leaving care, testing a model based on personal resources (self-esteem and self-efficacy) and formal and informal supports.  Young people at the end of their last year in care were included, and follow-ups were conducted for up to four years after leaving care.  At T1 questionnaires were conducted, phone interviews a year later (T2) and then interviews at T3 (following completion of military service).  Young people were provided a voucher as an incentive. | Mixed-methods adopted: quant to test a model for predicting life satisfaction and qual. to understand life stories and perspectives regarding life satisfaction.  Thematic analysis was adopted re: qualitative interviews.  The Israeli version of the Student’s Life Satisfaction Scale (SLSS), Self-esteem scale, Relationship with Father/Mother Questionnaire was used. And readiness for independent living questions were adopted, and the Medical Outcomes Study (MOS). | Life satisfaction was low - life satisfaction at one year post care was found to be a strong predictor of life satisfaction four years post care.  Personal resources contributed to subjective wellbeing. Self-efficacy contributed to life satisfaction and supports transitions.  Relationships like mentors, but especially birth family relationships enhanced life satisfaction.  Services focussed on independent living did not correlate with life satisfaction. |
| **23** | Kääriälä, Haapakorva [82]  Finland | Data is linked from different administrative records, for both children and their parents.  Used a comparison group of young people who had never been in care as well as the general population. | The study includes social, demographic, and health-related data for all children born in Finland in 1987.  12 employment and education adulthood trajectories. | On a population level OOHC experience was associated with temporal transitional trajectories in early adulthood, given their increased exposure to childhood adversities.  Young people with OOHC experience were less likely to have stable work and employment (38% compared to 62% of comparison group and 74% of the general population).  Young adults with care experience were more likely than peers never in care to have children in early adulthood.  OOHC experience and a higher number of placements increased the likelihood of entering early parenthood and fragmented unemployment and social assistance trajectories.  After-care support enhanced housing and employment trajectories. |
| **24** | Zeira, Refaeli [81]  Israel | The purpose is to test a model that predicts **Aspirations toward Higher Education (AtHE)** based on personal resources and the support from mothers and peers.  Study is linked to Refaeli, et al. (2019) above. | An exploratory study. Similar instruments as above used for data collection. The main difference is the focus on different measures: dependant variables relating to level of certainty in pursuing higher education, attitudes towards higher education, and amount of time and effort taken for planning higher education. Educational characteristics measured via: High-school achievement. | Academic experiences meant more AtHE, unlike vocational routes.  If high school is completed successfully, higher education is more likely.  Preparation for independent living, positive future thinking and educational self-efficacy positively correlated with AtHE. Self-esteem did not correlate. |
| **25** | Goyette and Blanchet [76]  Canada | The **EDJeP** study was a representative study from Canada. Panel data was used consisting of two waves of interviews and young people’s data from their administrative records was linked for analysis.  The focus was on living conditions and the exit from care of 17 - 21 year olds, their housing stability, education and career paths and personal difficulties in accessing social and health services. | Young people contacted were required to complete a questionnaire. Interviews were then conducted both over the phone and face-to-face.  Questions during interview focussed on experiences in OOHC and relationships with biological family.  Exploration of the intersection between biography and relations takes place to understand functioning post-care. | Data showed that young people who perceived they were supported were more successful. Tutoring for younger children and mentoring for older children was recommended.  Placement settings need to prioritise education and links are drawn between a stable placement and positive education outcomes.  Extended care was recommended as a means of support. And for young people who report homelessness were more likely to experience physical and psychological ill health. |
| **26** | Blakeslee, Miller [59]  USA | **Project Futures** is an adaptation of the My Life [35] study. It aimed to evaluate a self-determination coaching model with a view to increase postsecondary engagement and success among enrolled college students with foster care history and mental health challenges.  Participants were provided at least one hour of coaching every week across the academic year. Coaches were provided 4 days training around barriers to post-secondary success. | Project Futures was an on-campus program serving the university and two local colleges.  Participants completed ‘paper-and-pencil’ surveys at baseline, before coaching could start, at post-intervention (9 months later) and 6 months later.  Instruments used include: The AIR Self-Determination Scale, the Self-determination Score, the Career Decision Self-Efficacy-Short Form, the Career Goals Exploration, and the Youth Efficacy/Empowerment-Mental Health measure. | Findings validated that increasing self-determination as a mechanism supports transitions. Also, validation re: coaching models to develop self-determination.  Self-determination supports careers-related self-efficacy and exploration. Campus support helps care experienced students, especially re: mental health and wellbeing.  Need for flexible programming that meets the multidimensional needs of these students. And there should be greater support and similar programs should be offered to high school students. |
| **27** | Zeira, Refaeli [71]  Israel | The convenience sample consisted participants from 26 residential care settings in Israel.  Work status explored at each T as well as source of income/financial situation. | Work status, source of income, importance of job economic hardships, and financial situation were themes addressed in questions at various time points. Measures at T1 focussed on work experiences prior to leaving care. At T2, 3, and 4 2 questions were asked in relation to experiences after ‘aging out’ of care. | T1 = 84.1% had worked at some point.  T2 = 27.3% were in work.  T3 = 98.6% had worked at some point (note most completed military service) and 83.9% were currently working.  T4 = 83.4% were working.  Employment offers an immediate response to economic needs. Employment status was positive – economic stability less so. Work experience is a central component of an individual's human capital, allowing them to acquire new skills and to develop other work-related competences.  Ten years after ageing out of care, when the participants were in their late 20s, the vast majority had a steady full-time job in which they wanted to continue for the coming years. Moreover, from a few years post-care on (at T3 and T4) their main source of income was salary. Experiences of debt 39.1% at T1 and 44.4% at T4. |
