# Supplementary material for: Understanding how young people transitioning from out-of-home care acquire and develop independent living skills and knowledge: A systematic review of longitudinal studies
Source: PLoS One. 2024 Jun 11;19(6):e0304965. doi: 10.1371/journal.pone.0304965 (PMC11166282; doi:10.1371/journal.pone.0304965)
Supplement: S4 Table — (DOCX) [file pone.0304965.s005.docx]

Fig 6: Methodological quality of included studies

|  | **Study** | **Rater agreement** | **Quality** |
| --- | --- | --- | --- |
| **1** | Cook [60] | 80% | Strong quality |
| **2** | Courtney, Piliavin [74] | 80% | Strong quality |
| **3** | Rashid [66] | 95% | Strong quality |
| **4** | Courtney, Dworsky [73] | 95% | Strong quality |
| **5** | Pecora, Williams [63] | 75% | Good quality |
| **6** | Uzoebo, Kioko [79] | 73% | Good quality |
| **7** | Senteio, Marshall [68] | 91% | Strong quality |
| **8** | Vorhies, Glover [70] | 91% | Strong quality |
| **9** | Van Ryzin, Mills [80] | 68% | Fair quality |
| **10** | Kirk and Day [77] | 90% | Strong quality |
| **11** | Powers, Geenen [35] | 85% | Strong quality |
| **12** | Lee, Courtney [64] | 100% | Strong quality |
| **13** | Sulimani-Aidan, Benbenishty [69] | 73% | Good quality |
| **14** | Hasson, Reynolds [65] | 86% | Strong quality |
| **15** | Greeson, Garcia [61] | 96% | Strong quality |
| **16** | Tyrell and Yates [78] | 95% | Strong quality |
| **17** | Dickens [28] | 85% | Strong quality |
| **18** | Fowler, Marcal [75] | 90% | Strong quality |
| **19** | Schwartz-Tayri and Spiro [67] | 80% | Strong quality |
| **20** | Hedin [62] | 85% | Strong quality |
| **21** | Boddy, Bakketeig [72] | 70% | Good quality |
| **22** | Refaeli, Benbenishty [45] | 95% | Strong quality |
| **23** | Kääriälä, Haapakorva [82] | 95.5% | Strong quality |
| **24** | Zeira, Refaeli [81] | 90% | Strong quality |
| **25** | Goyette and Blanchet [76] | 85% | Strong quality |
| **26** | Blakeslee, Miller [59] | 80% | Strong quality |
| **27** | Zeira, Refaeli [71] | 95% | Strong quality |
